# Study on development of accessory sex glands in prepubertal kids using two-dimensional ultrasonography

**DOI:** 10.14202/vetworld.2016.346-350

**Published:** 2016-04-06

**Authors:** Sonu Kumari, R. A. Luthra, R. K. Chandolia, Sandeep Kumar, Parveen Kumar, Devender, Ankit Kumar, Nidhi Bishnoi, Sunil Bishnoi

**Affiliations:** 1 Department of Veterinary Gynaecology and Obstetrics, Lala Lajpat Rai University of Veterinary & Animal Sciences, Hisar - 125 004, Haryana, India; 2 Department of Veterinary Medicine, Lala Lajpat Rai University of Veterinary & Animal Sciences, Hisar - 125 004, Haryana, India; 3 Department of Veterinary Pathology, Lala Lajpat Rai University of Veterinary & Animal Sciences, Hisar - 125 004, Haryana, India; 4 Department of Livestock Products Technology, Lala Lajpat Rai University of Veterinary & Animal Sciences, Hisar - 125 004, Haryana, India

**Keywords:** accessory sex glands, kids, prepubertal, ultrasonography

## Abstract

**Aim:**

The present study was undertaken to study growth pattern of accessory sex glands in prepubertal kids from 2 weeks to 6 months of age using two-dimensional ultrasonography.

**Materials and Methods:**

The study was conducted on six Beetal kids. The scanning of accessory sex glands was done in standing position using rectal probe and measurements were recorded. Data collected were statistically analyzed using one-way ANOVA followed by Duncan multiple range test was performed using the SPSS (16.0) system for windows.

**Results:**

With the advancement of age all the dimensions of glands increased. Both the lobes of prostate gland showed an increase in width with advancement of age. Width of prostate above the urethra (W_1_) showed a significant increase at 2, 10, and 20 weeks of age, whereas non-significant increase from 2 to 8, 10 to 19, and 20 to 24 weeks of age was recorded. Width of prostate below the urethra (W_2_) showed a significant increase at 20 weeks of age, whereas non-significant increase was recorded during rest of period of growth. Left and right bulbourethral gland showed a similar pattern of growth with the advancement of age. The circumference dimensions increased significantly at 2, 16, 20, and 21 weeks of age for both glands. The increase was non-significant from 4 to 14, 16 to 19, and 20 to 23 weeks of age. The same pattern was observed for left and right seminal vesicular gland.

**Conclusion:**

Significant growth in three accessory sex glands in prepubertal kids was not observed at the same age. The trend observed was that the prostate was the first gland to show significant growth at 10 weeks of age followed by a significant increase in seminal vesicles and bulbourethral gland at 14 and 16 weeks of age, respectively.

## Introduction

Ultrasonography is a non-invasive diagnostic method and examination can be carried out quickly and easily [1]. Ultrasonography is based on the ability of reflection of transmitted high-frequency sound waves by tissue. Ultrasound waves are generated by the piezoelectric effect in a suitable medium such as lead zirconate. Echoes of ultrasound depend on relative density of tissue [2]. A, B, and M modes are the three basic forms of ultrasound used in soft tissue imaging. A-mode ultrasonic imaging is a one-dimensional display of echo amplitudes versus distance. B-mode ultrasonic imaging produces an accurate two-dimensional (2D) cross-sectional image of soft tissues. M-mode ultrasonic imaging is an adaptation of B-modes to evaluate moving structure of the heart. In this study, B-mode ultrasonography was used.

Diagnostic ultrasound frequencies range from 2 to 15 MHz and are inaudible to the human ear. The ultrasonogram is essentially an image of a thin slice of tissue, and therefore, enables serial examinations to monitor the progression of the condition, response to treatment and to practice scanning techniques [3]. However, one of the drawbacks of diagnostic ultrasound is that it requires a great deal of skill and experience. In diagnostic ultrasound tissue interfaces can be detected, and their shape and size can be determined. Ultrasonography is a valuable alternative imaging system that can provide more accurate information about reproductive disorders in comparison to traditional methods [4]. Ultrasound is used in females for pregnancy diagnosis, reproductive disorders [5-7] similarly its use in male animal reproduction is gaining importance for diagnosis of various disorders [8].

Buck has three accessory sex glands, *viz*., seminal vesicles, prostate, and bulbourethral glands. Disorders of accessory sex glands are of diagnostic importance but till now less work has been done on ultrasonography of buck accessory glands. Previously by different authors ultrasonography of the accessory sex glands was done in other species, i.e., in bull [9,10], horse [11], dog [12], ram [13], etc. but no reports are there in prepubertal kids. Hence, the study was planned to observe the development of the accessory sex glands before puberty and to provide a basic data of the glands to help in diagnostics of various diseases related to accessory sex glands.

## Materials and Methods

### Ethical approval

No invasive method was used in the present study.

### Place of study

The study was conducted in Department of Veterinary Gynaecology and Obstetrics, College of Veterinary Sciences, Lala Lajpat Rai University of Veterinary and Animal Sciences (LUVAS), Hisar.

### Animals

Six healthy Beetal kids of 2 weeks of age were selected and kept for study till 6 months of age. The study was conducted from November till May. They were kept on grazing as well stall feeding.

### Ultrasonographic examination

**Figure 1:**
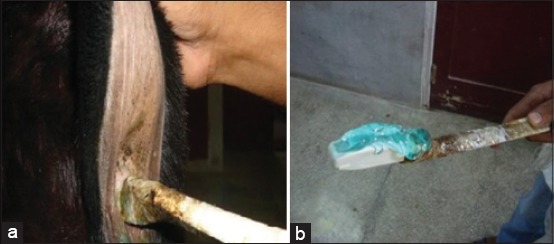
(a) Pre-rectal approach for scanning accessory sex glands in kids, (b) two-dimensional curvilinear trans-rectal probe used for study modified by polyvinyl chloride pipe and adhesive tape for per-rectal approach.

The ultrasonography of accessory sex glands was done in standing position using per-rectal probe (Figure-1a). No sedation was given to animals. The ultrasound machine used for this study was three-dimensional ultrasound machine (Nemio-XG: Toshiba, Japan) having four-dimensional volumetric probe. 2D intraoperative probe of this machine having frequencies between 5 and 10 MHz was made stiff by fixing it on a 0.5” polyvinyl chloride pipe after slitting it and using adhesive tape (Figure-1b). It was used for transrectal ultrasonography. Scanning of glands was done 2 weeks apart from 2 to 16 weeks of age and weekly after that till 24 weeks of age. The landmark for seminal vesicle is urinary bladder. It is present at the neck of bladder as irregular gland. The prostate is present on both sides of urethra. Bulbourethral gland is scanned as a round gland near the ischial arch. The measurements of accessory glands were recorded.

### Analysis of the images and interpretation of data

The ultrasound images recorded in the machine were reviewed in the scanner itself to re-examine the images in detail. The data collected were statistically analyzed for finding out average mean and standard error. Differences at a p<0.05 were considered to be statistically significant. One-way ANOVA followed Duncan multiple range test were performed using the SPSS (16.0) system for windows.

## Results

**Figure 2:**
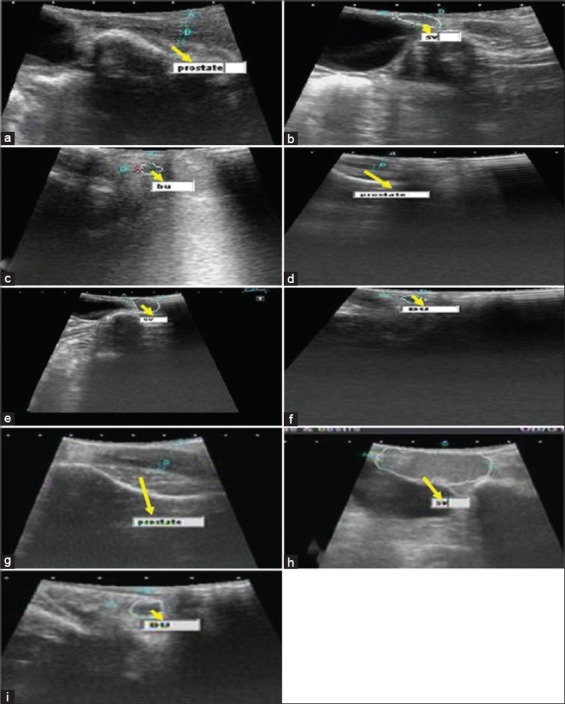
(a) Ultrasound image of the prostate gland at 2 weeks of age (yellow arrow), (b) ultrasound image of the seminal vasicular gland (sv) at 2 weeks of age (yellow arrow), (c) ultrasound image of the bulbourethal gland (bu) at 2 weeks of age (yellow arrow), (d) scanning of prostate gland at 16 weeks of age (yellow arrow), (e) scanning of sv gland at 16 weeks of age (yellow arrow), (f) scanning of bu gland at 16 weeks of age (yellow arrow), (g) scanned image of prostate gland at 24 weeks of age (yellow arrow), (h) scanned image of sv gland at 24 weeks of age (yellow arrow), (i) scanned image of bu gland at 24 weeks of age (yellow arrow).

2D ultrasonography was done for the study of accessory sex glands. At 2 weeks of age, the prostate gland was observed surrounding the pelvic urethra as two disseminated lobes (Figure-2a). The prostatic dimensions (W_1_ and W_2_) were 3.55±0.21 and 5.45±0.37 mm (Table-1), respectively. Seminal vesicles (Figure-2b) were observed as paired, smooth, and lobular gland dorsolateral to the neck of urinary bladder having a circumference of left and right gland as 21.03±0.66 and 21.07±0.68 mm (Table-2), respectively. Bulbourethral gland (Figure-2c) was also observed as a paired gland located on either side of pelvic urethra near the point where the pelvic urethra emerge from the pelvis region. The gland is closely related to the root of penis and was oval in shape and pea-sized. The left and right bulbourethral gland circumference dimensions were 10.93±0.38 and 10.85±0.37 mm (Table-3), respectively. With advancement of age the dimensions of the glands increased along with an increase in the echogenicity of the glands.

**Table-1:** Average mean±SE of the width of prostate gland (n=6).

Age in weeks	W_1_ (mm)	W_2_ (mm)
2	3.55^a^±0.21	5.45^a^±0.37
4	3.58^a^±0.21	5.57^ab^±0.35
6	3.70^a^±0.21	5.65^abc^±0.34
8	3.78^a^±0.22	5.72^abc^±0.35
10	5.03^b^±0.22	6.43^abcd^±0.15
12	5.30^b^±0.18	6.52^abcd^±0.17
14	5.35^b^±0.16	6.60^abcd^±0.16
16	5.45^b^±0.17	6.75^bcd^±0.17
17	5.51^b^±0.18	6.83^cd^±0.21
18	5.66^b^±0.17	7.00^d^±0.25
19	5.75^b^±0.24	7.15^d^±0.25
20	7.65^c^±0.31	8.52^e^±0.63
21	7.66^c^±0.31	8.57^ef^±0.62
22	8.10^c^±0.45	8.60^ef^±0.37
23	8.31^c^±0.37	9.70^fg^±0.53
24	8.36^c^±0.16	9.88^g^±0.58

Means with different superscript within the column are different significantly (p<0.05). SE=Standard error

**Table-2:** Average mean±SE of the circumference (C) of left and right seminal vesicular gland (n=6).

Age in weeks	C (mm) (left)	C (mm) (right)
2	21.03^a^±0.66	21.07^a^±0.68
4	26.22^a^±2.43	26.20^a^±2.44
6	26.65^a^±2.40	26.68^a^±2.41
8	29.78^a^±2.43	29.87^a^±2.41
10	31.08^a^±2.48	31.07^a^±2.48
12	41.23^b^±1.20	41.28^b^±1.21
14	48.10^b^±1.13	48.03^b^±1.15
16	66.42^c^±4.77	66.45^c^±4.77
17	66.52^c^±4.74	66.45^c^±4.75
18	66.58^c^±4.76	66.57^c^±4.75
19	66.67^c^±4.74	66.70^c^±4.74
20	73.15^cd^±3.23	73.10^cd^±3.22
21	74.37^cd^±2.68	74.38^cd^±2.68
22	74.55^cd^±2.71	74.53^cd^±2.71
23	75.08^cd^±2.82	75.12^cd^±2.84
24	80.85^d^±4.37	80.80^d^±4.37

Means with different superscript within the column are different significantly (p<0.05). SE=Standard error

**Table-3:** Average mean±SE of the circumference (C) of left and right bulbourethral gland (n=6).

Age in weeks	C (mm) (left)	C (mm) (right)
2	10.93^a^±0.38	10.85^a^±0.37
4	12.78^ab^±0.31	12.81^ab^±0.31
6	14.33^bc^±0.35	14.30^bc^±0.35
8	15.45^bc^±0.27	15.50^bc^±0.26
10	15.53^bc^±0.27	15.50^bc^±0.26
12	16.15^bc^±0.63	16.15^bc^±0.62
14	17.51^c^±0.68	17.56^c^±0.68
16	21.33^d^±0.47	21.36^d^±0.46
17	21.78^d^±0.56	21.73^d^±0.56
18	22.01^d^±0.57	21.98^d^±0.57
19	23.76^d^±0.59	23.73^d^±0.59
20	29.93^e^±1.19	29.96^e^±1.18
21	29.98^e^±1.18	30.00^e^±1.19
22	30.18^e^±1.24	30.15^e^±1.24
23	30.45^e^±1.17	30.41^e^±1.16
24	37.31^f^±3.37	37.36^f^±3.35

Means with different superscript within the column are different significantly (p<0.05). SE=Standard error

Both the lobes of prostate gland showed an increase in width with the advancement of age. W_1_ showed a significant increase at 2, 10, and 20 weeks of age, whereas non-significant increase from 2 to 8, 10 to 19 and 20 to 24 weeks of age was recorded. W_2_ showed a significant increase at 20 weeks of age, whereas non-significant increase was recorded during rest of period of growth. Left and right bulbourethral gland showed a similar pattern of growth with advancement of age. The circumference dimensions increased significantly at 2, 16, 20 and 21 weeks of age for both glands. The increase was non-significant from 4 to 14, 16 to 19 and 20 to 23 weeks of age. The same pattern was observed for left and right seminal vesicular gland. At 16 weeks of age there was an increase in the echogenicity of the glands along with increase in dimensions of all the glands. W_1_ and W_2_ prostatic dimensions were 5.45±0.17 and 6.75±0.17 mm (Figure-2d), left and right seminal vesicle were 66.42±4.77 and 66.45±4.77 mm (Figure-2e), whereas left and right bulbourethral gland dimensions were found to be 21.33±0.47 and 21.36±0.46 mm (Figure-2f), respectively.

The dimensions of all the three glands increased and at 24 weeks of age, the prostatic dimensions (Figure-2g) (W_1_ and W_2_) were 8.36±0.16 and 9.88±0.58 mm (Table-1), seminal vesicles (Figure-2h) were 80.85±4.37 and 80.80±4.37 mm (Table-2), bulbourethral gland (Figure-2i) dimensions were 37.31±3.37 and 37.36±3.35 mm (Table-3), respectively.

## Discussion

The development defects or pathological conditions can cause infertility/sterility in male animals by affecting spermatozoa production and maturation process [14]. Moreover, the available basic data on the ultrasonographic developmental characteristics of accessory sex glands in male goats is also very scanty.

Three accessory sex glands are present in buck. Seminal vesicle gland is the major gland and is a paired lobular gland present dorsolateral to the neck of urinary bladder [15-20]. Prostate gland not so well developed in bucks, was observed as two disseminated lobes surrounding the pelvic urethra [15]. The bulbourethral gland was observed as a paired gland, oval in shape and pea sized and located on either side of pelvic urethra near the point where the pelvic urethra emerge from the pelvis region [16].

The accessory sex glands were scanned per-rectally in standing position using a technique described in rams [21,22]. A 7.5 MHz transducer was used which provided better details of the glands. In the early phase of development (2-6 weeks of age), all the glands were anechoic, this indicated an active phase of fluid accumulation in these glands [21]. With advancement of age the echogenicity of glands increased, this was attributed to cell and connective tissue proliferation in the glands [21].

In the present study seminal, vesicle gland was visualized as an anechoic lobulated and irregular gland at 2 weeks of age and as age advanced the echogenicity increased. Left and right seminal vesicles were almost of equal circumference as reported by Khalaf and Merhish [16] in the anatomical study of glands. The increase in circumference of the gland was gradual from 2 weeks till 14 weeks of age followed by a rapid increase in dimension from 14 to 16 weeks of age. Thereafter, the increase was gradual and again a steep increase was observed from 23 to 24 weeks of age. There are no parallel studies in goat, however, in ram the segmental increase has been attributed to the early rise in testosterone around 3 months and increased levels around 4 months of age [21].

The dimensions of prostate gland in the present study increased with the advancement of age. The width of both lobes increased gradually with age. The width of lobe above the urethra (W_1_) was having lesser width than the lobe below the urethra (W_2_). The significant change in the dimensions of the prostate gland was at two-time points, i.e. between 8 and 10 weeks of age and 19^th^ to 20^th^ week of age. This can be attributed to the level of circulating testosterone as has been reported previously in ram [21]. In that study, early rise in testosterone has been shown around 10 weeks of age and major change after 4 months.

In the present study, bulbourethral gland was observed as a paired gland. The circumference of both the glands was equal in dimension and similar growth pattern was recorded. There was gradual increase in circumference of the gland with rapid increase observed between 14 and 16 weeks of age and then at 20 and 24 weeks of age of development. Since similar data is not available in the literature on buck, but this can be attributed to increase in testosterone level in ram as reported previously in ram [21].

## Conclusion

The seminal vesicle, prostate, and the bulbourethral are the three accessory sex glands present in bucks. Seminal vesicle gland is the major gland and is a paired lobular gland present dorsolateral to the neck of urinary bladder. Ultrasonographically, urinary bladder was visible as anechoic and seminal vesicle glands were visible echoic in relation to urinary bladder. Both the seminal vesicle gland were irregular, lobulated and knobbed structure. The circumference of both the glands was almost equal, i.e. both develop equally with advancement of age. As age advanced the echogenicity of the gland increased, the visibility of the prostate gland was clear from the 6 weeks onward. The prostatic parenchyma was observed to have a coarse echogenicity. Ultrasonographically, bulbourethral gland was observed as oval and pea sized and was present on either side of pelvic urethra near the point where the pelvic urethra emerges from the pelvis region. The trend in growth observed in buck was that prostate was the first gland to show significant growth at 10 weeks of age then significant increase was observed in seminal vesicles at 14 weeks of age and then in bulbourethral gland at 16 weeks of age.

## Authors’ Contributions

SK, RAL and RKC proposed the study. SK, PK, DD and SB carried out the ultrasonography and management of the plan of study. NB and SK critically observed the data and calculates mean and used SPSS statistical software. AK, SK and SK prepared the manuscript. Finalization of manuscript was done by RAL, RKC and SK. All authors read and approved the final manuscript.
